# Synthesis and Nonisothermal Crystallization Kinetics of Poly(Butylene Terephthalate-*co*-Tetramethylene Ether Glycol) Copolyesters

**DOI:** 10.3390/polym12091897

**Published:** 2020-08-24

**Authors:** Hsu-I Mao, Chin-Wen Chen, Syang-Peng Rwei

**Affiliations:** Institute of Organic and Polymeric Materials, Research and Development Center of Smart Textile Technology, National Taipei University of Technology, No. 1, Sec. 3, Chung-Hsiao East Road., Taipei 10608, Taiwan; eeric0212@hotmail.com

**Keywords:** poly(butylene terephthalate), poly(tetramethylene ether glycol), copolymer, nonisothermal crystallization

## Abstract

Poly(butylene terephthalate-*co*-tetramethylene ether glycol) (PBT-*co*-PTMEG) copolymers with PTMEG ranging from 0 to 40 wt% were synthesized through melt polymerization. The structure and composition were supported by Fourier-transform infrared spectroscopy (FT-IR) and nuclear magnetic resonance spectroscopy (^1^H NMR). All samples had excellent thermal stability at a T_d−5%_ around 370 °C. Crystallization temperature (T_c_) and enthalpy of crystallization (ΔH_c_) were detected by differential scanning calorimetry (DSC), revealing a decrement from 182.3 to 135.1 °C and 47.0 to 22.1 J g^−1^, respectively, with the increase in PTMEG concentration from 0 to 40 wt%. Moreover, nonisothermal crystallization was carried out to explore the crystallization behavior of copolymers; the crystallization rate of PBT reduced gradually when PTMEG content increased. Hence, a decrement in the spherulite growth rate was detected in polarizing light microscope (PLM) observation, observing that the PTMEG could enhance the hindrance in the molecular chain to lower the crystallinity of PBT-*co*-PTMEG copolyester. Moreover, thermal properties and the crystallization rate of PBT-*co*-PTMEG copolymers can be amended via the regulation of PTMEG contents.

## 1. Introduction

Thermoplastic poly(ether ester) elastomer (TPEE) [1], as a member of thermoplastic elastomers (TPE), is widely used in the market [2,3,4,5,6]. The synthesis of TPEE was first reported by Coleman [7] in 1954, and TPEE was described to be a block copolymer consisting of two separated phases: Crystalline aromatic polyester as a hard segment [8,9,10,11,12,13,14,15] and amorphous polyether as a soft segment [16,17,18]. The microphase separation [19] can give the TPEE excellent mechanical properties [17,20,21,22] in high-temperature performance, oil resistance, solvent resistance from the rigidity, polarity, and crystallinity from a hard segment, low-temperature performance, and aging resistance from the soft segment [23,24]. Hence, the mechanical properties of TPEE could be tuned via adjusting the ratio of the hard segment and soft segment. Poly(butylene terephthalate) (PBT) [25,26,27,28,29,30], which possesses excellent mechanical properties, thermal stability, thermal processability, and prominent crystallization ability, is seen as one of the most considerable hard segment materials.

PBT-based TPEE has been developed, such as poly(butylene terephthalate-*co*-polyethylene glycol) (PBT-*co*-PEG) [16,31] and poly(butylene terephthalate-*co*-polypropylene glycol) (PBT-*co*-PPG) [32], revealing varied characteristics depending on different compositions. Poly(butylene terephthalate-*co*-tetramethylene ether glycol) (PBT-*co*-PTMEG) copolymer was first commercialized under the trademark “Hytrel” (Du pont, Wilmington, DE, United States) as engineering plastics in 1972 [33,34,35], and the morphology [17,36,37,38,39,40,41,42,43,44], properties [45,46,47,48,49], interactions between hard segments and soft segments [50,51], and structure of the crystalline [36,38,52,53,54,55,56,57,58] and amorphous phase [5,17,49,59] have been studied extensively. The crystalline regions of PBT-*co*-PTMEG copolymers are continuous and highly interconnected, dispersing in a continuous amorphous region [52,60]. Similar to styrene-butadiene-styrene (SBS), where the glassy domain consisting of styrene blocks provides the physical crosslinking network [61,62], crystallized hard segments were connected by covalent bonds as a physical crosslinking point. When stress is exerted on the copolymer, the stress is gradually transferred from the crystalline phase to the elastomeric portions of the polymer network.

Furthermore, the amorphous phase of copolymers is microphase separated into a PBT/PTMEG mixed-phase and a highly mobile PTMEG-rich phase with a relatively large concentration of soft segments and relatively long soft-segment block lengths [17]. The higher the content of the soft segment, the greater the reinforcement of elasticity achieved [63]. However, the increase in soft segment content results in a reduction in crystallization ability [64], which is unfavorable for the cases of high-speed processing such as injection molding and ultrahigh-speed spinning [65,66,67,68,69].

A series of PBT-*co*-PTMEG copolymers with different ratios of PTMEG to PBT segments were synthesized to analyze the influence on crystallization ability with PTMEG proportion, and the thermal properties were determined using differential scanning calorimetry in this research. The effect of PTMEG concentration in various cooling rates was analyzed and discussed using nonisothermal crystallization. The crystallization kinetics of copolymers was studied and compared to Avrami [70], Mo [71], and Kissinger [72] models. Moreover, the spherulite morphology and crystal growth rate were inspected by polarized light microscopy (PLM).

## 2. Experimental

### 2.1. Materials

Pure terephthalic acid (PTA, 98%), 1,4-butanediol (1,4-BDO, 99%), polytetramethylene ether glycol (PTMEG, 99%) with a number-average molecular weight (M_n_) of 2000 g/mol, and titanium(IV) butoxide (Ti(OBu)_4_, 97%) as catalysts were purchased from Sigma-Aldrich (St. Louis, MO, USA). All the materials were adopted in melting polymerization without purification.

### 2.2. Synthesis of PBT-co-PTMEG Copolymers

**Scheme 1:**
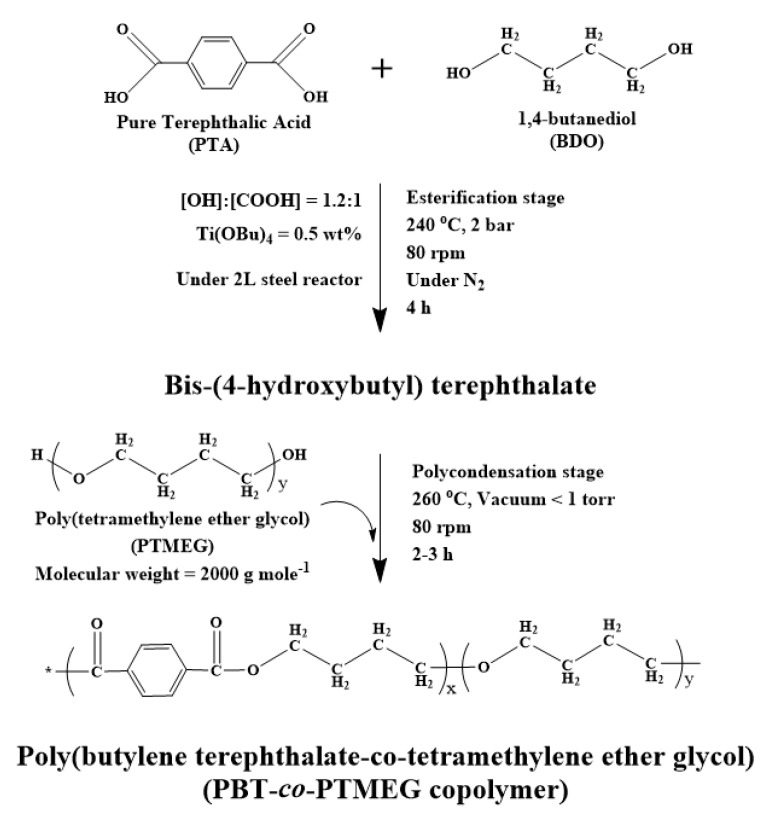
Synthesis route of poly(butylene terephthalate-*co*-tetramethylene ether glycol) copolymers.

PBT-*co*-PTMEG copolymers were copolymerized with a two-step strategy via bulk polymerization. PTMEG was separately added after PBT prepolymer was obtained. Scheme 1 describes the detailed process of the synthesis. In the first step, a mixture of PTA, 1,4 BDO, and the catalyst was placed in an autoclave under vacuum and then purged with N_2_ gas. This cycle was repeated three times. The mixture was heated to 240 °C under N_2_ until the amount of the distilled water reached 95% of its theoretic amount in order to obtain the PBT prepolymer. In the second step, the second portion of the catalyst and various amounts of PTMEG were added in the system. The system was slowly heated up to 250 °C under 1 kPa pressure and maintained for 1 h. After this, the temperature was raised and held at 260 °C under a high vacuum below 1.0 Torr. When the torque value reached a certain amount, copolymerization was selected to finish the reaction. Finally, the product was cooled in iced water for further analysis.

### 2.3. Measurement

#### 2.3.1. Nuclear Magnetic Resonance Spectroscopy (^1^H-NMR)

Structure and compositions of PBT-*co*-PTMG were determined by a JEOL ECZ600R ^1^H-NMR spectrometer. Trifluoroacetic acid (TFAAC) was used as a solvent. The measurement was performed at 25 °C, and there were 128 recorded scans.

#### 2.3.2. Fourier-Transform Infrared Spectroscopy (FT-IR)

FTIR spectrometer (PerkinElmer, Waltham, MA, USA) was employed to identify the synthesized PBT-co-PTMEG copolymers in attenuated total reflection mode with an average signal of 32 co-added scans at a resolution of 4 cm^–1^ over a wavenumber range of 400–4000 cm^−1^.

#### 2.3.3. Intrinsic Viscosity (I.V.)

Intrinsic viscosity (I.V.) of PBT-*co*-PTMEG copolymers was measured by a Ubbelohde viscometer. The solvent used for the measurement was a phenol/tetrachloroethane mixture (50/50, wt%), and the concentration was 1.0 g dL^−1^. The system was kept at 25.0 ± 0.1 °C in a water bath. The viscosity average molecular weight (M_n_) of each sample was calculated using the Mark–Houwink equation:

$$\left[\eta \right]=K{\left[M_{n}\right]}^{\alpha }$$

where *K* = 1.7 * 10^−4^, *α* = 0.83.

#### 2.3.4. Differential Scanning Calorimetry (DSC)

DSC (Hitachi High Tech. DSC-7000, Tokyo, Japan) was employed to measure the thermal properties of PBT-*co*-PTMEG copolymers to analyze the melting temperature (T_m_) and crystallization temperature (T_c_), as well as melting enthalpy (ΔH_m_) and crystallization enthalpy (ΔH_c_). All the samples were prepared in 5 mg and dried in the oven at 80 °C for one day. Each experiment was kept in an atmosphere of nitrogen (20 mL min^−1^) in an aluminum pan. PBT-*co*-PTMEG copolymers were discovered from 50 to 240 °C at a heating rate of 10 °C min^−1^ and kept at 240 °C for 5 min to remove the thermal history. Next, these PBT-*co*-PTMEG copolymers were cooled to 50 °C at a cooling rate of 10 °C min^−1^ for the first round of cooling. Finally, the second round of heating was performed from 50 to 240 °C at the same heating rate of 10 °C min^−1^ to obtain the T_m_. By doing so, the values of T_c_ and T_m_ were exhibited from the exothermic peak and endothermic peak through the first round of cooling and the second round of heating procedures, respectively.

#### 2.3.5. Thermogravimetric Analysis (TGA)

TGA (Hitachi, STA 7200, Tokyo, Japan) was adopted to measure the degradation temperature of the synthesized PBT-*co*-PTMEG copolymers. The synthesized samples in the weight range of 5–10 mg were heated from 50 to 700 °C at a heating rate of 10 °C min^−1^ under a nitrogen atmosphere. The degradation temperature from the TGA curve was determined at 5% weight loss (T_d-5%_).

#### 2.3.6. X-ray Diffraction (XRD)

The sample was first melted at 240 °C and flattened at a pressure of 50 Kgf cm^−2^ for 3 min, and then cooled at 10 °C min^−1^ to room temperature. Wide-angle X-ray scattering was carried out by a Malvern Panalytical X’Pert^3^ powder diffractometer (Malvern, UK) with Cu K_α_ radiation (λ = 0.154 nm) in 2θ from 10 to 40° at room temperature with a scanning speed of 0.2° min^−1^.

#### 2.3.7. Nonisothermal Crystallization Analysis

DSC (Hitachi High Tech. DSC-7000, Tokyo, Japan) was employed to measure the thermal properties of PBT-*co*-PTMEG copolymers. All the samples were prepared in 5 mg and dried in the oven at 80 °C for one day. Each experiment was kept in an atmosphere of nitrogen (20 mL min^−1^) in an aluminum pan. The sample was first heated from 50 to 240 °C at 10 °C min^−1^ and then kept for 5 min to remove the thermal record. Then, it was reduced to 50 °C with the different rates of 2, 5, 10, and 20 °C min^−1^, and held for 5 min. The heat flow curves were recorded to observe the nonisothermal crystallization behavior.

#### 2.3.8. Polarizing Light Microscope (PLM)

PLM (Nikon, ECLIPSE LV100N POL, Tokyo, Japan) was equipped with a Linkam THMS Examina / FTIR600 heating stage, a Linkam ECP water cooling control unit, and a Nikon camera with the NIS Elements imaging software. The heating temperature ranged from 50 to 240 °C at a rate of 150 °C min^−1^ and held for 5 min to erase the thermal history, and was then rapidly cooled to the target crystallization temperature at a cooling rate of 150 °C min^−1^. Subsequently, a set temperature was held for 30 min to observe the crystallization growth rate, and the crystal morphology and growth of PBT-*co*-PTMEG copolymers were taken by PLM video under isothermal crystallization conditions.

## 3. Results and Discussion

**Figure 1:**
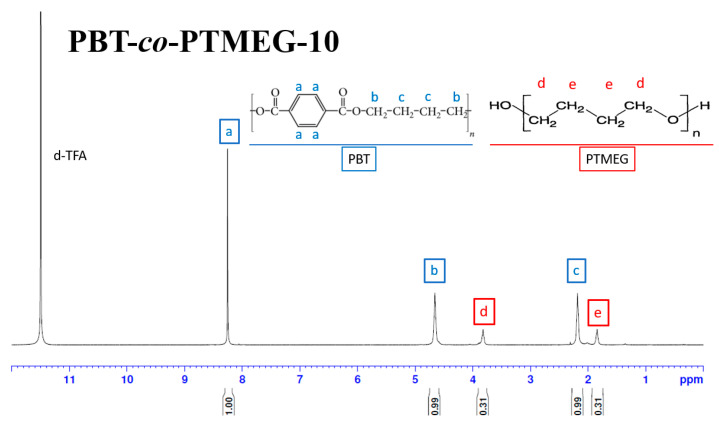
^1^H NMR spectrum of poly(butylene terephthalate-*co*-polypropylene glycol) (PBT-*co*-PTMEG)-10 copolymer.

Figure 1 shows the chemical structure, and the comonomer composition of PBT-*co*-PTMEG -10 copolymer, as observed by ^1^H-NMR spectroscopy [40]. Resonance peaks of PBT-*co*-PTMEG copolymers were identified and assigned in: Peak a (8–8.5 ppm) corresponds to the aromatic protons of the terephthalate units. Peak b and peak c (4–4.5 and 2–2.5 ppm) are ascribed to the proton signals of O(CH_2_)_4_O in the PBT segment. Peak d and peak e (3.5–4 and 1.5–2 ppm) are ascribed to the proton signals of O(CH_2_)_4_O in the PTMEG segment. Chemical structures of PBT-*co*-PTMEG copolymers were confirmed. The composition of PTMEG within PBT copolymers can be calculated by Equation (1):

(1) $$w_{PTMEG}\left(\mathrm{wt}\%\right)=\frac{72\times \left(I_{d}/4\right)}{220\times \left(I_{a}/4\right)+72\times \left(I_{d}/4\right)}\times 100\%$$

where 220 and 72 are the molecular weight of the PBT and the PTMEG repeat units, respectively. *I_a_* and *I_d_* are the integral areas of the “a” peak and “d” peak, respectively. The intrinsic viscosity, number average molecular weight (M_n_), weight ratio based on ^1^H-NMR, and theory of the copolymers are shown in Table 1.

**Figure 2:**
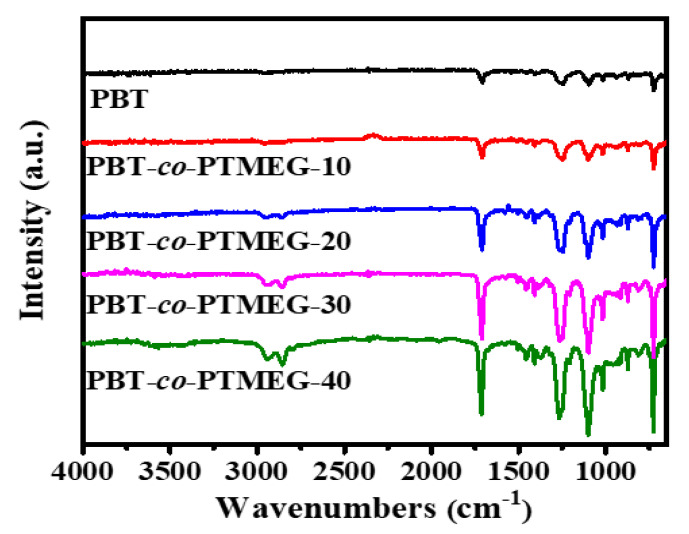
FT-IR spectra of PBT and PBT-*co*-PTMEG copolymers.

Figure 2 displays the FT-IR spectra of synthesized PBT-*co*-PTMEG copolymers, the characteristic absorption peaks related to the (CH_2_)_4_ of ether-ester at 727 cm^−1^, the stretching vibration of the -COO- absorption of the PBT ester group at 1274 cm^−1^, C=O of the PBT ester group at 1713 cm^−1^, the C-O-C backbone vibration of PTMEG at 1099 cm^−1^, and the -CH_2_- stretch vibration of PTMEG at 2861 and 2944 cm^−1^ [49]. The intensity of corresponding peaks was enhanced with the increase in PTMEG concentration, suggesting that PBT-*co*-PTMEG copolymers were synthesized successfully.

**Figure 3:**
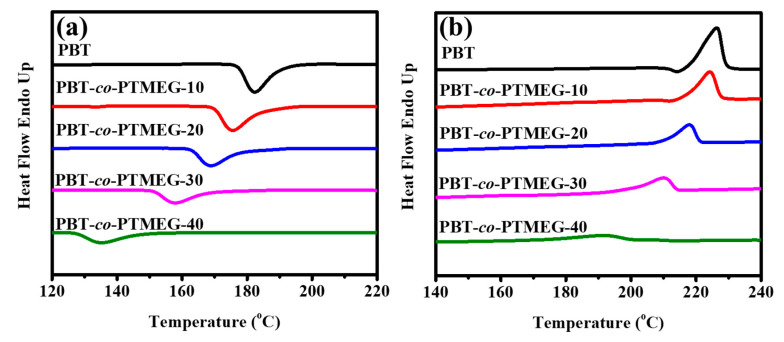
DSC curves of PBT and PBT-*co*-PTMEG copolymers in (**a**) 1st cooling process and (**b**) 2nd heating process at a rate of 10 °C min^−1^.

DSC curves of the first cooling process displayed in Figure 3a and the 2nd heating process in Figure 3b demonstrated that T_c_ decreased with the increase in PTMEG content from 182.3 to 135.1 °C for PBT and PBT-*co*-PTMEG-40, and a broader crystallization peak was observed, suggesting that the incorporation of PTMEG hindered the PBT regular chain from packing into an ordered state, which results in a lower T_c_ and ΔH_c_. As expected, the T_m_ and overall ΔH_m_ were also reduced due to the crystallization region of the PBT-rich domain being disrupted by the PTMEG segments. All the thermal properties are summarized in Table 2.

**Figure 4:**
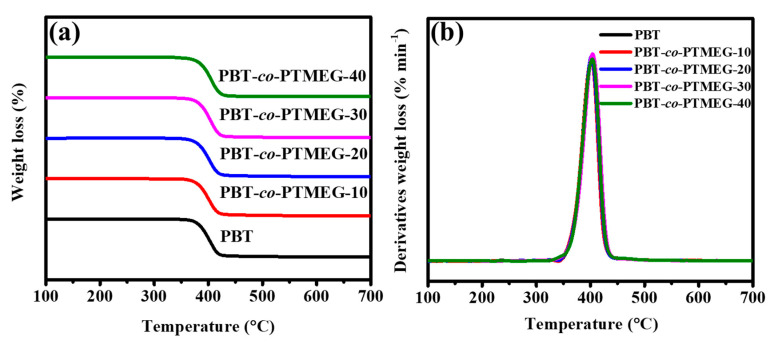
(**a**) Weight loss and (**b**) derivative thermogravimetry (DTG) as a function of temperature for PBT and PBT-*co*-PTMEG copolymers.

Figure 4a,b display the thermal decomposition and derivatives weight loss curves for PBT-*co*-PTMEG copolymers. All samples show a single-step decomposition track in the whole process. The 5% weight loss temperature (T_d-5%_) was located at around 369 to 371 °C. In other research, a reduction in thermal stability may be found while a high content of soft segment exists, which makes the T_d-5%_ drop significantly [63]. The comparable value of T_d-5%_ for PBT-*co*-PTMEG copolymers, indicating excellent thermal stability, was maintained while being copolymerized with PTMEG into PBT below 40 wt%.

**Figure 5:**
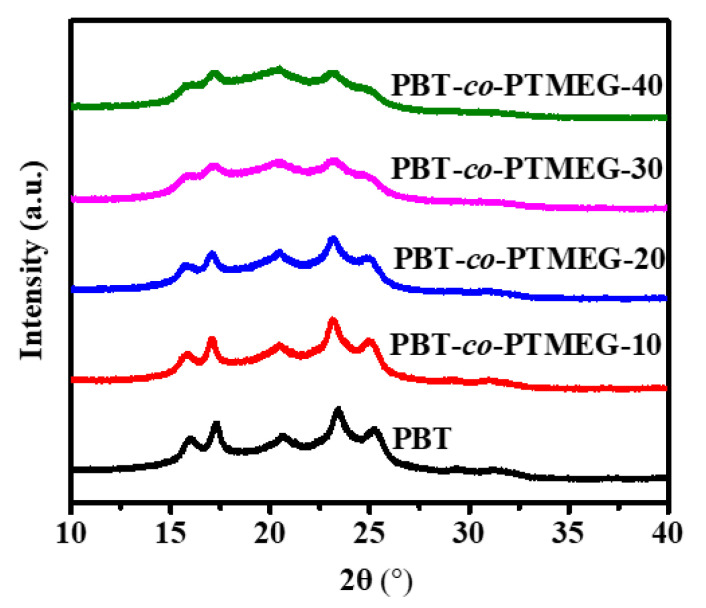
XRD patterns of PBT and PBT-*co*-PTMEG copolymers.

Figure 5 presents the XRD results of PBT-*co*-PTMEG copolymers in a 2θ range of 10–40°. Five characteristic peaks of XRD of PBT-*co*-PTMEG copolymers were carried out around the 2θ values of 15.8°, 17.1°, 20.4°, 23.1°, and 25.0° for the crystal lattices of $\left(0\bar{1}0\right),\;\left(010\right),\;\left(011\right),\;\left(100\right),\;\mathrm{and}\;\left(1\bar{1}1\right)$, respectively, which corresponded to the α-form as neat PBT, demonstrating no phase transition from α to β on PBT domains with the incorporation of PTMEG [30,46,56,58,60,65,73] despite some changes in the peak intensity. The attenuation of intensity, related to a reduction in crystallinity, could be explained by the fact that the incorporation with PTMEG would limit crystallization due to the disruption of the PBT regular chain, where this result exhibits an excellent relationship by DSC analysis.

**Figure 6:**
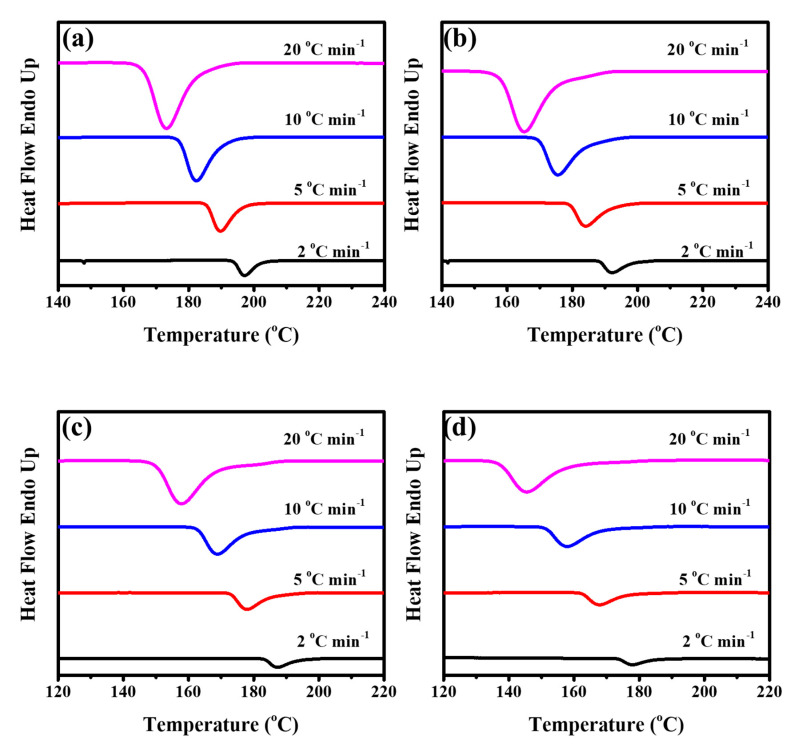

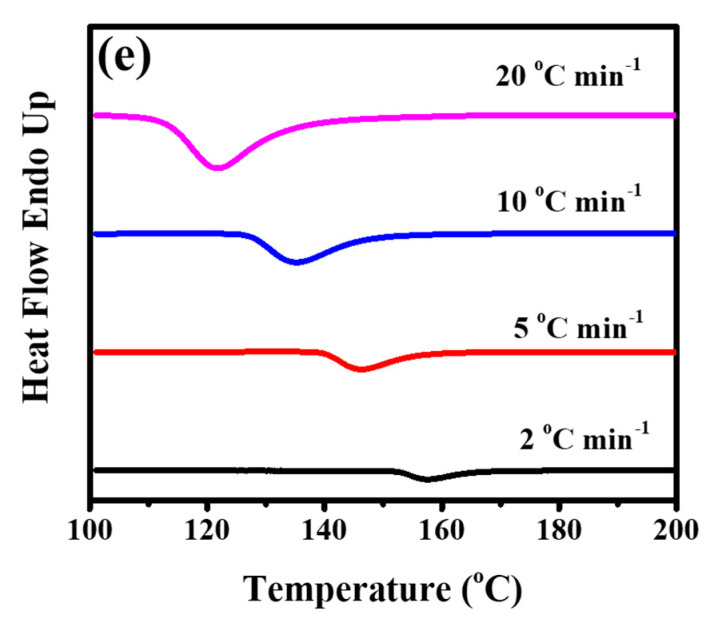
DSC curves of PBT-*co*-PTMEG copolymers in cooling process at 2, 5, 10, and 20 °C min^−1^: (**a**) PBT, (**b**) PBT-*co*-PTMEG-10, (**c**) PBT-*co*-PTMEG-20, (**d**) PBT-*co*-PTMEG-30, and (**e**) PBT-*co*-PTMEG-40.

Figure 6 displays nonisothermal DSC curves of PBT-*co*-PTMEG copolymers in a temperature range from 50 to 240 °C under 2, 5, 10, and 20 °C min^−1^. The exothermic peak becomes more extensive than the PBT and shifts to a lower temperature as the cooling rate increases, demonstrating that the motion of the molecular chains is not fast enough to attain thermal equilibrium under a high cooling rate [74]. Besides, an exothermic event as a shoulder of the peak on the higher temperature side was observed when PTMEG was added, implying a microphase separation of PBT-rich and PTMEG-rich phases. With the temperature decreased, the PBT-rich phase crystallized at a higher temperature range, and PTMEG-rich phases crystallized successively at a lower temperature, resulting in a “shoulder” shape with a peak, especially at a higher cooling rate.

The Avrami model [70] is the most common way to describe the overall crystallization kinetics:

(2) $$X\left(t\right)=1-\exp\left(-kt^{n}\right)$$

**Figure 7:**
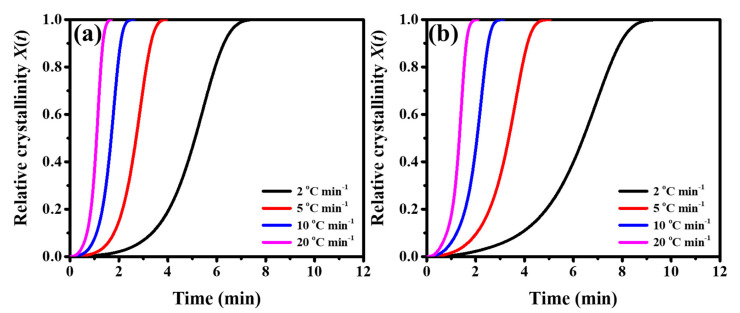

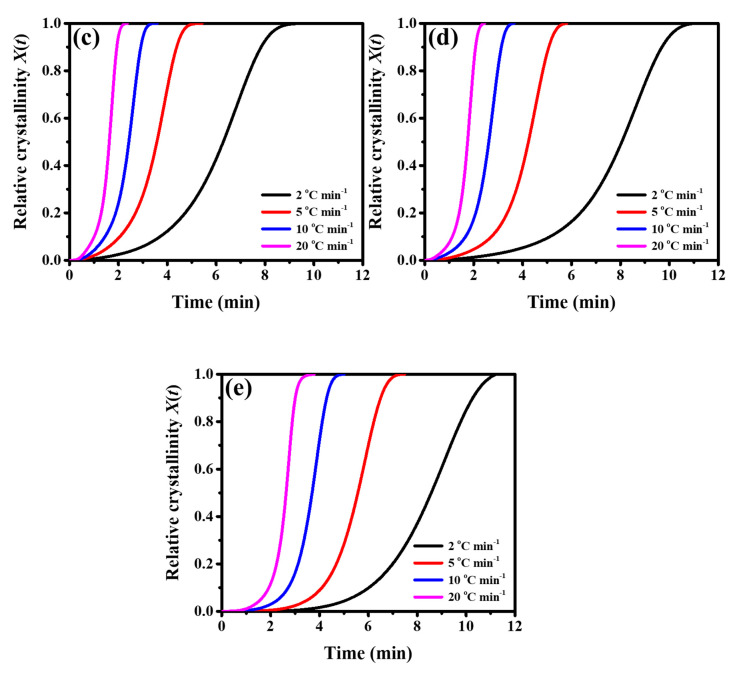
Relative crystallinity (*X*(*t*)) as a function of crystallization time at various temperatures for PBT-*co*-PTMEG copolymers at different contents of PTMEG. For synthesized copolymer, (**a**) PBT, (**b**) PBT-*co*-PTMEG-10, (**c**) PBT-*co*-PTMEG-20, (**d**) PBT-*co*-PTMEG-30, and (**e**) PBT-*co*-PTMEG-40 at various cooling rates. The solid lines represent the DSC experimental results of the Avrami equation.

(3) log{−ln[1−x(t)]}=−logk+nlogt

where *X*(*t*) is the relative crystallinity fraction at different crystallization times *t*, *n* is the Avrami exponent, and *k* is the crystallization rate constant. The value of n contains information on the nucleation mechanism and growth geometry. The relative crystallinity fraction *X*(*t*) can be determined by the following equation:

(4) $$X\left(t\right)=\frac{X_{C}\left(t\right)}{X_{C}\left(t_{\infty }\right)}=\frac{\int_{0}^{t}\frac{dH\left(t\right)}{dt}dt}{\int_{0}^{\infty }\frac{dH\left(t\right)}{dt}dt}$$

where *X_c_*(*t*) and *X_c_*(*t*_∞_) are the heat generated at time t and infinite time *t*_∞_, respectively, and d*H*(*t*)/d*t* is the rate of heat flow, which means that d*H*(*t*) is the enthalpy of crystallization at a given temperature during the time interval dt via DSC measurement. The relative crystallinity fraction *X*(*t*) as a function of time was calculated. The result is shown in Figure 7.

**Figure 8:**
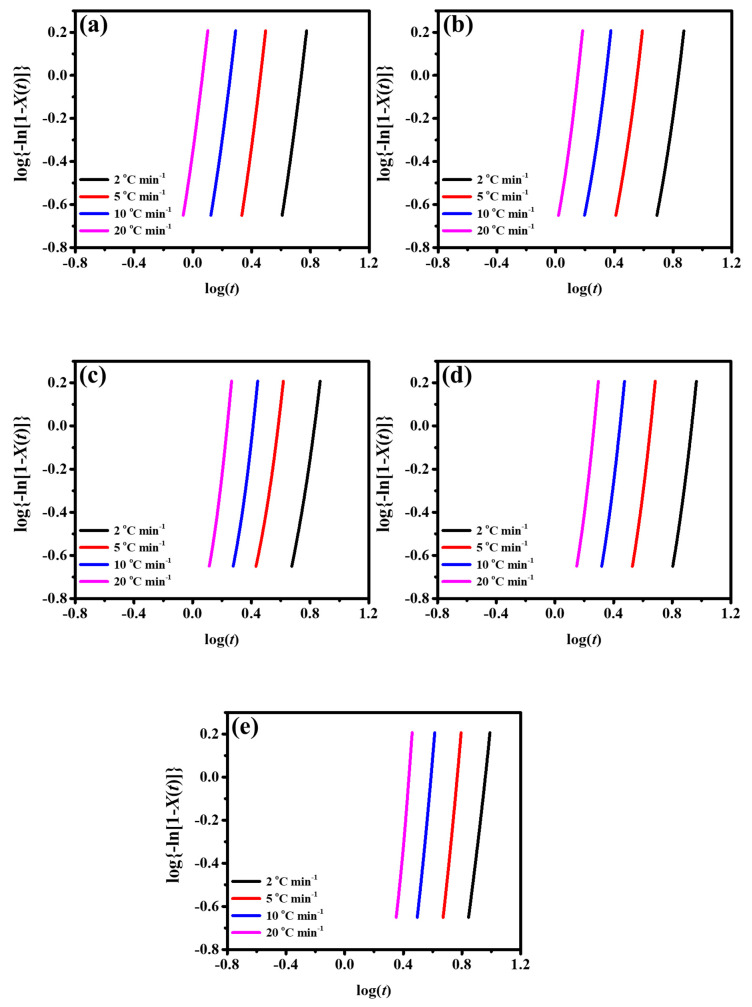
Avrami plots for PBT-*co*-PTMEG copolymers at different PTMEG contents of (**a**) PBT, (**b**) PBT-*co*-PTMEG-10, (**c**) PBT-*co*-PTMEG-20, (**d**) PBT-*co*-PTMEG-30, and (**e**) PBT-*co*-PTMEG-40 at various cooling rates.

The curve of log{–ln[1–*X*(*t*)]} versus log *t* at the *X*(*t*) in a range from 20 to 80% was obtained, as presented in Figure 8. The Avrami exponent *n* and the crystallization rate constant *k* were obtained from the slope and the intercept of the curve. The half-time and growth rate were also obtained using the following equation:

*t*_1/2_ = (ln2/*k*)^(1/^*^n^*^)^ (5)

*G* = 1/*t*_1/2_ (6)

All the data are summarized in Table 3. The Avrami exponent *n* of all samples was in the range between 4.5 and 7.9. According to the theory of the Avrami model, the value of *n* should be located between 1 and 4, suggesting a failure in the prediction of crystal structure, and the deviation of *n* (*n* > 4) was occasionally observed in nonisothermal crystallization kinetics analysis [28]. The crystallization rate constant *k* and the growth rate *G* values of PBT-*co*-PTMEG copolymers indicate that a lower crystallization rate was obtained with a higher content of PTMEG. The trend is similar to T_c_, as discussed previously, meaning the crystallization ability of PBT was reduced with the incorporation of PTMEG. It could be explained that the PTMEG disrupted regulation of the PBT molecular chain, which hindered the crystallinity of the copolymer.

**Figure 9:**
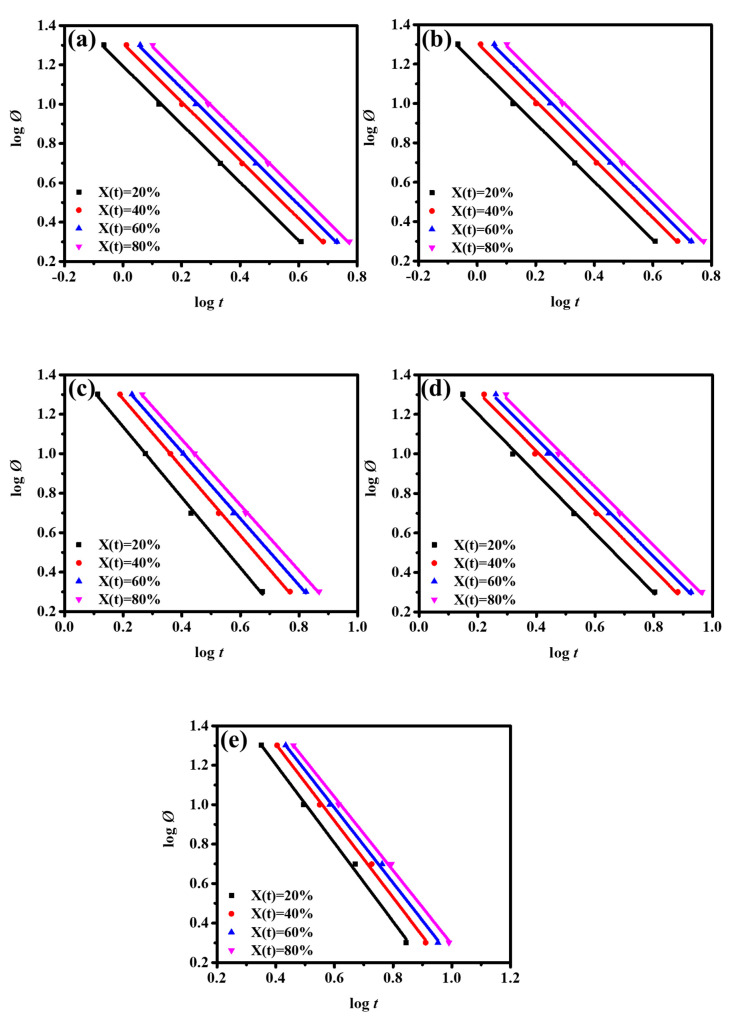
Curves of log ∅ versus log *t* for PBT-*co*-PTMEG copolymers: (**a**) PBT, (**b**) PBT-*co*-PTMEG-10, (**c**) PBT-*co*-PTMEG-20, (**d**) PBT-*co*-PTMEG-30, and (**e**) PBT-*co*-PTMEG-40.

The Mo model [71] is also one of the most common methods to analyze the nonisothermal crystallization process as the following equation:

(7) log ∅=log F(T)−a log t

where a is Mo’s exponent, *a* = (*n*/*m*). *F*(*T*) is a parameter of cooling rate in which the system reaches a certain degree of crystallinity in unit time. A smaller value of *F*(*T*) reflecting the higher crystallization rate can be achieved. The curve of log ∅ versus log *t* was obtained and is presented in Figure 9. A good linear relationship was performed in all samples, revealing that this method is suitable to describe the nonisothermal crystallization process of these systems. Mo’s exponent and *F*(*T*) can be calculated from the slope and the intercept of the curves. All data are tabulated in Table 4. The value of *F*(*T*) increased with the ratio of PTMEG in the copolymer at a given relative crystallinity, indicating the incorporation with PTMEG decreasing the crystallization rate of PBT-*co*-PTMEG copolymers. This result is in good accordance with the Avrami model analysis.

**Figure 10:**
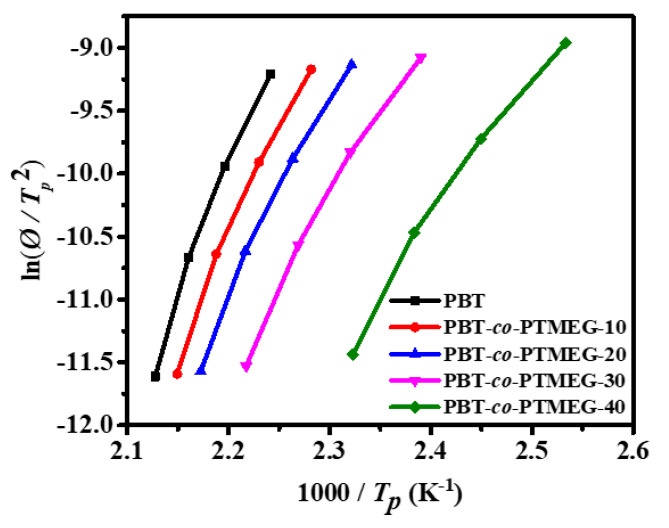
Plots of ln(∅/*T_p_*^2^) versus 1000/*T_p_* for PBT and PBT-*co*-PTMEG copolymers.

Activation energy is also an important index to determine the crystallization ability of copolymers, which is related to the energy required for the transport of crystalline chains across the inter-phase [75]. The Kissinger model [72] is almost a universal method in the calculation of activation energy, which is based on the variation in the peak of crystallization temperature with cooling rate ∅:

(8) $$\left[\ln\left(\emptyset /Tp^{2}\right)\right]/d\left(1/T_{p}\right)=\left(-\Delta E\right)/R$$

where Δ*E* is the activation energy, *T_p_* is the peak temperature of crystallization, and *R* is the universal gas constant. The curves of ln(∅/*T_p_*^2^) versus 1000/*T_p_* are displayed in Figure 10, and the value of Δ*E* was issued from the slope. The ΔE was detected at the lowest value for neat PBT and increased with the PTMEG ratios (−173.04, −150.05, −134.38, −117.05, and −96.64 kJ for PBT, PBT-*co*-PTMEG-10, PBT-*co*-PTMEG-20, PBT-*co*-PTMEG-30, and PBT-*co*-PTMEG-40, respectively). This demonstrates that the incorporation of PTMEG into PBT enhanced the energy barrier of copolymers, revealing a reduction in the crystallization rate due to the irregular chain. This result agrees well with the trend observed previously by Avrami and Mo’s model.

Spherulitic morphology and dimension could affect the physical properties of copolymers [76]. However, it is difficult to observe the whole nonisothermal crystallization process due to the high crystallization rate of PBT-*co*-PTMEG copolymers. Isothermal crystallization of copolymers was investigated under a PLM with a hot stage and cooling system to study the spherulitic morphology and growth process visually.

**Figure 11:**
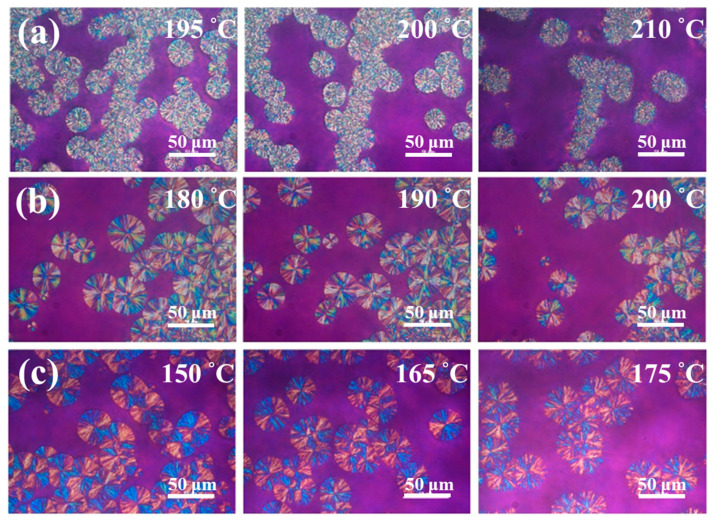
Polarizing light microscopy (PLM) image of PBT-*co*-PTMEG at the selected temperature: (**a**) PBT (**b**) PBT-*co*-PTMEG-10, and (**c**) PBT-*co*-PTMEG-40.

The composition of copolymers plays an important role, which may have a bearing on the spherulite growth rate and morphology. Images of PBT-*co*-PTMEG copolymers at a selected temperature are presented in Figure 11 and Figure S2. A similar morphology of the negative spherulite with a Maltese cross [77] was observed in all samples. Additionally, crystallization temperature also has a key role in driving the growth of spherulitic morphology. The nucleation density of copolymers was reduced as the isothermal crystallization temperature increased, attributed to the greater difficulty of nucleation. Furthermore, the swifter growth of spherulites at a relatively low temperature was surveyed.

Theoretically, a low temperature is favorable for nucleation; however, the mobility of the molecular chain was more restricted, which causes relatively concentrated spherulites with a low growth rate. By contrast, at high temperatures, the nucleation becomes more unstable and vulnerable due to the movement of the molecular chain to increase drastically; only small and few spherulites were formed. In summary, the highest spherulitic growth rate can be acquired only at a temperature range favoring both nucleation and crystal growth [76]; between these two extremes, the growth rate passes through a maximum where the two factors are approximately equal in magnitude [77].

**Figure 12:**
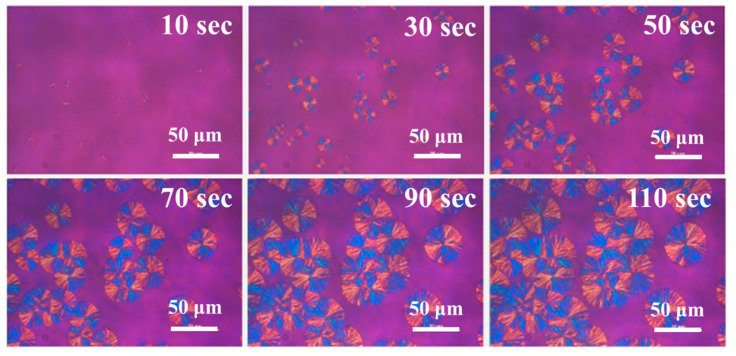
Spherulite morphology of PBT-*co*-PTMEG-40 copolymer at 160 °C as a function of time.

However, the observation of spherulitic growth rate isothermally at all temperatures is difficult due to the high crystalline speed of PBT-*co*-PTMEG copolymers, and the spherulite overgrows during the cooling process. Therefore, only the process at higher temperatures was surveyed completely. Taking PBT-*co*-PTMEG-40 as an example, the spherulitic growth process under a specific temperature (160 °C) at a different time is displayed in Figure 12. The growth rate of spherulites (*G*) can be obtained by recording the variation in the spherulitic dimension.

**Figure 13:**
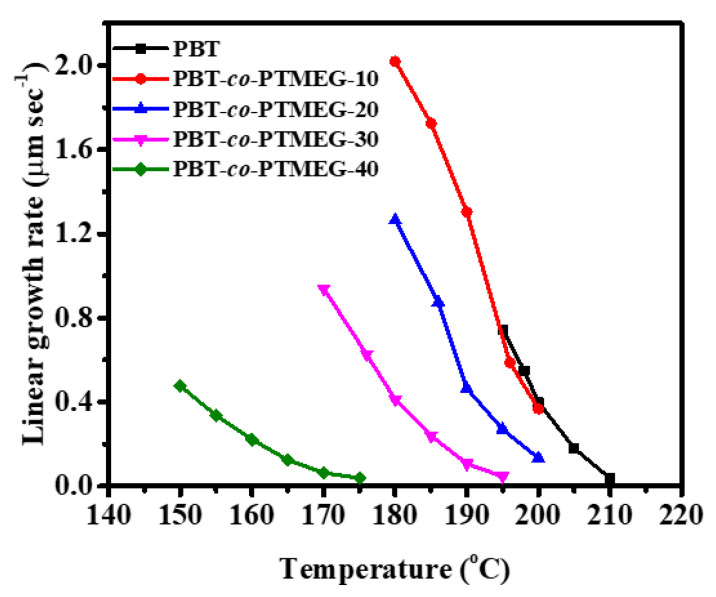
Spherulite growth rates as a function of crystallization temperature for PBT and PBT-*co*-PTMEG copolymers.

Spherulite growth rates as a function of T_c_ for PBT-*co*-PTMEG copolymers were calculated, as presented in Figure 13. It can be seen that PBT-*co*-PTMEG-10 shows a comparable value of *G* as neat PBT. Nevertheless, the *G* value of PBT-*co*-PTMEG copolymers decreased significantly as the content of PTMEG increased more. The accumulation of the soft segment can play two roles: (1) Promoting crystallization due to the enhancement of flexibility of the hard segment chain, which is commonly observed in low-crystallization-rate systems such as PET; (2) hindering crystallization by the disruption of the PBT regular chain. When 10 wt% PTMEG is employed for PBT, no notable variety appears on the growing rate of copolymers, due to the low content of the soft segment and high crystallization ability of PBT. When more PTMEG is employed, the effect of hindrance becomes more durable due to the increasing irregular molecular chain structure; therefore, the crystallization ability decreases dramatically, which display a similar tendency from DSC results discussed previously.

## 4. Conclusions

A series of PBT-*co*-PTMEG copolymers with PTMEG ranging from 0 to 40 wt% were synthesized via melt polymerization. The structure and composition were designated by ^1^H-NMR and FT-IR. PBT-*co*-PTMEG copolymers retain excellent thermal stability at a T_d-5%_ around 370 °C. However, the T_c_ and ΔH_c_ decreased from 182.3 to 135.1 °C and 47.0 to 22.1 J g^−1^ with PTMEG content from 0 to 40 wt%, indicating a reduction in crystallization ability. The characteristic peaks of XRD were detected around the 2θ values of 15.8°, 17.1°, 20.4°, 23.1°, and 25.0° for the crystal lattices of $\left(0\bar{1}0\right),\;\left(010\right),\;\left(011\right),\;\left(100\right),\;\left(1\bar{1}1\right)$, respectively, which corresponded to the α-form as neat PBT. Moreover, a weakening of peaks in intensity was observed with the addition of PTMEG, revealing an excellent agreement to DSC results. The crystallization rate was measured by nonisothermal crystallization kinetics analysis. The higher PTMEG ratio within PBT-*co*-PTMEG copolymers can directly affect the crystallization rate, which decreases with the increase in the PTMEG content due to the disruption of the PBT regular chain. A hindrance of crystallization was explored, and a similar tendency was observed in the Avrami, Mo, and Kissinger model. The spherulite growth rate of copolymers was compared using PLM; a comparable value of spherulite growth rate *G* was found in PBT-*co*-PTMEG-10 as neat PBT, which then decreased significantly as the content of PTMEG increased more, suggesting a rising effect of the hindrance.

## Figures and Tables

**Table 1:** Characteristic data of PBT and PBT-*co*-PTMEG copolymers.

Sample	I.V.	M_n_	*w_PTMEG_*−Feed	*w_PTMEG_*−NMR
	(dL g^−1^)	(g mole^−1^)	(wt%)	(wt%)
PBT	0.99	34,384	0	0
PBT-*co*-PTMEG-10	0.90	30,654	10	9.1
PBT-*co*-PTMEG-20	0.85	28,614	20	17.5
PBT-*co*-PTMEG-30	1.16	41,618	30	28.5
PBT-*co*-PTMEG-40	1.33	49,073	40	41.8

**Table 2:** Thermal properties of PBT and PBT-*co*-PTMEG copolymers.

Sample	T_c_	ΔH_c_	T_m_	ΔH_m_	T_d-5%_
	(°C)	(J g^−1^)	(°C)	(J g^−1^)	(°C)
PBT	182.3	47.0	226.3	51.4	371.5
PBT-*co*-PTMEG-10	175.5	45.9	224.7	49.8	369.6
PBT-*co*-PTMEG-20	168.8	37.5	218.6	39.2	369.7
PBT-*co*-PTMEG-30	157.8	28.6	209.4	31.6	370.5
PBT-*co*-PTMEG-40	135.1	22.1	190.5	24.3	370.0

**Table 3:** Avrami analysis for nonisothermal crystallization and half-time of crystallization for PBT and PBT-*co*-PTMEG copolymers at different PTMEG contents.

Sample	*Ø*	*n*	*k*	*t_½_*	*G*
	(°C min^−1^)	-	(min^−n^)	(min)	(min^−1^)
PBT	2	5.2	1.48 × 10^−4^	5.09	0.196
	5	5.3	3.58 × 10^−3^	2.69	0.372
	10	5.1	4.91 × 10^−2^	1.67	0.599
	20	5.2	4.57 × 10^−1^	1.08	0.926
PBT-*co*-PTMEG-10	2	4.7	1.21 × 10^−4^	6.35	0.157
	5	4.7	2.32 × 10^−3^	3.33	0.300
	10	4.8	2.26 × 10^−2^	2.04	0.490
	20	5.2	4.57 × 10^−1^	1.35	0.741
PBT-*co*-PTMEG-20	2	4.5	2.00 × 10^−4^	6.23	0.161
	5	4.6	1.99 × 10^−3^	3.54	0.282
	10	5.2	7.30 × 10^−3^	2.41	0.415
	20	5.7	4.47 × 10^−2^	1.61	0.621
PBT-*co*-PTMEG-30	2	5.3	1.05 × 10^−5^	8.00	0.125
	5	5.6	2.37 × 10^−4^	4.21	0.238
	10	5.6	8.66 × 10^−3^	3.52	0.284
	20	5.9	2.71 × 10^−2^	1.74	0.575
PBT-*co*-PTMEG-40	2	5.9	2.33 × 10^−6^	8.54	0.117
	5	7.0	4.37 × 10^−6^	5.55	0.180
	10	7.2	5.71 × 10^−5^	3.68	0.272
	20	7.9	3.30 × 10^−4^	2.62	0.382

**Table 4:** Nonisothermal kinetic parameters for PBT and PBT-*co*-PTMEG copolymers obtained by Mo’s equation.

Sample		Relative Crystallinity (%)			
		20	40	60	80
PBT	*F*(*T*)	11.99	15.59	18.28	21.20
	*a*	1.47	1.48	1.48	1.48
PBT-*co*-PTMEG-10	*F*(*T*)	20.51	27.22	31.64	35.95
	*a*	1.47	1.46	1.45	1.44
PBT-*co*-PTMEG-20	*F*(*T*)	31.11	41.94	48.38	54.40
	*a*	1.78	1.72	1.68	1.66
PBT-*co*-PTMEG-30	*F*(*T*)	31.99	41.10	47.06	52.85
	*a*	1.51	1.50	1.49	1.48
PBT-*co*-PTMEG-40	*F*(*T*)	100.04	122.09	133.72	144.17
	*a*	1.99	1.94	1.90	1.86

## Supplementary Materials

The following are available online at https://www.mdpi.com/2073-4360/12/9/1897/s1.
