# Supplementary material for: Synthesis and Nonisothermal Crystallization Kinetics of Poly(Butylene Terephthalate-co-Tetramethylene Ether Glycol) Copolyesters
Source: Polymers (Basel). 2020 Aug 24;12(9):1897. doi: 10.3390/polym12091897 (PMC7565969; doi:10.3390/polym12091897)
Supplement: Supplementary file 1 [file polymers-12-01897-s001.pdf]

Supporting Information

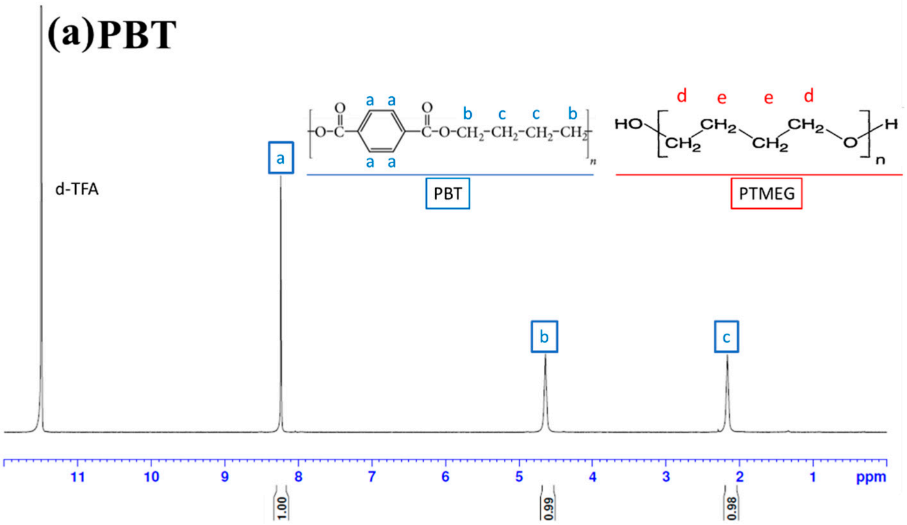

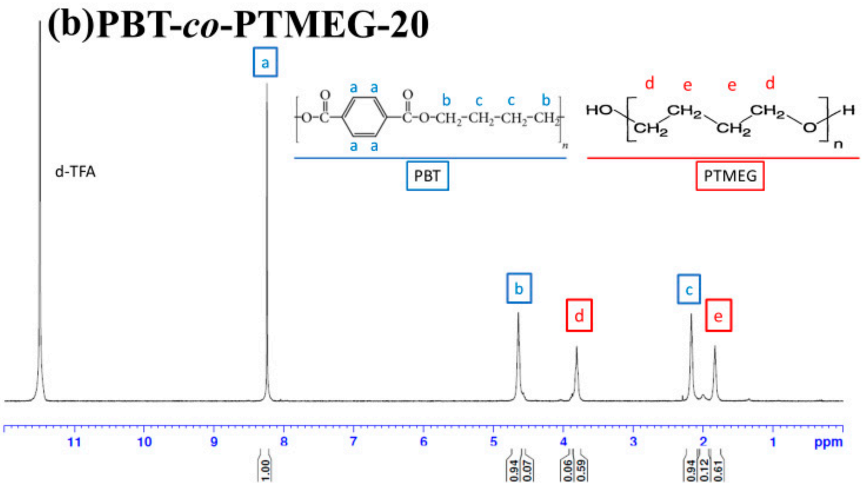

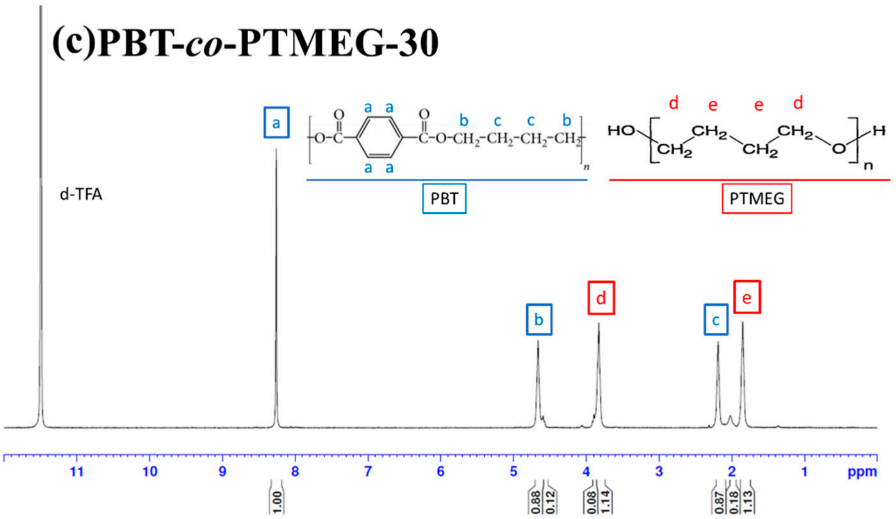

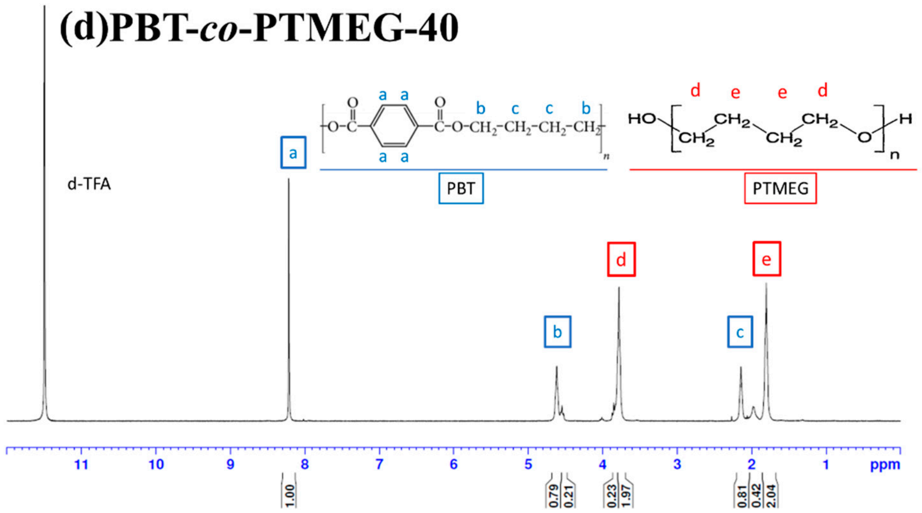

Figure S1. ^{1}H NMR spectrum of copolymers (a) PBT (b) PBT-*co*-PTMEG-20 (c) PBT-*co*-PTMEG-30 (d) PBT-*co*-PTMEG-40.

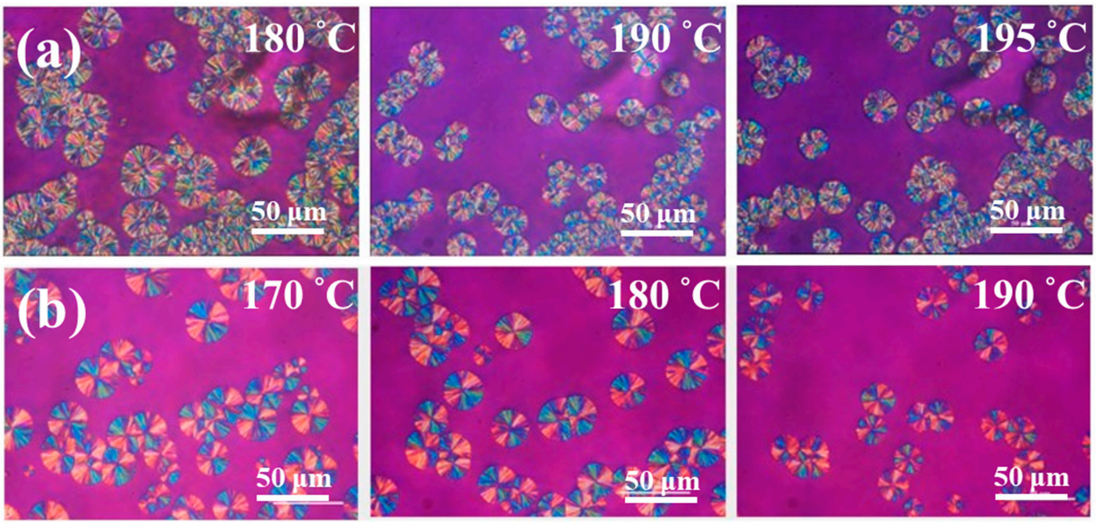

Figure S2. PLM image of PBT-*co*-PTMEG at the selected temperature (a) PBT-*co*-PTMEG-20 (b) PBT-*co*-PTMEG-30.
